# DDIAS promotes STAT3 activation by preventing STAT3 recruitment to PTPRM in lung cancer cells

**DOI:** 10.1038/s41389-019-0187-2

**Published:** 2020-01-02

**Authors:** Joo-Young Im, Bo-Kyung Kim, Kang-Woo Lee, So-Young Chun, Mi-Jung Kang, Misun Won

**Affiliations:** 1 Personalized Genomic Medicine Research Center, KRIBB, Daejeon, 34141 Korea; 2 Department of Oriental Medicine, Daejeon University, Daehak-ro 62, Dong-gu, Daejeon, 34520 South Korea; 3 Department of Functional Genomics, KRIBB School of Bioscience, Korea University of Science and Technology (UST), 217 Gajeong-ro, Yuseong-gu, Daejeon, 34113 Korea

**Keywords:** Tumour biomarkers, Extracellular signalling molecules, Non-small-cell lung cancer, Phosphorylation, Oncogenes

## Abstract

DNA damage-induced apoptosis suppressor (DDIAS) regulates cancer cell survival. Here we investigated the involvement of DDIAS in IL-6–mediated signaling to understand the mechanism underlying the role of DDIAS in lung cancer malignancy. We showed that DDIAS promotes tyrosine phosphorylation of signal transducer and activator of transcription 3 (STAT3), which is constitutively activated in malignant cancers. Interestingly, siRNA protein tyrosine phosphatase (PTP) library screening revealed protein tyrosine phosphatase receptor mu (PTPRM) as a novel STAT3 PTP. PTPRM knockdown rescued the DDIAS-knockdown-mediated decrease in STAT3 Y705 phosphorylation in the presence of IL-6. However, PTPRM overexpression decreased STAT3 Y705 phosphorylation. Moreover, endogenous PTPRM interacted with endogenous STAT3 for dephosphorylation at Y705 following IL-6 treatment. As expected, PTPRM bound to wild-type STAT3 but not the STAT3 Y705F mutant. PTPRM dephosphorylated STAT3 in the absence of DDIAS, suggesting that DDIAS hampers PTPRM/STAT3 interaction. In fact, DDIAS bound to the STAT3 transactivation domain (TAD), which competes with PTPRM to recruit STAT3 for dephosphorylation. Thus we show that DDIAS prevents PTPRM/STAT3 binding and blocks STAT3 Y705 dephosphorylation, thereby sustaining STAT3 activation in lung cancer. DDIAS expression strongly correlates with STAT3 phosphorylation in human lung cancer cell lines and tissues. Thus DDIAS may be considered as a potential biomarker and therapeutic target in malignant lung cancer cells with aberrant STAT3 activation.

## Introduction

Lung cancer is a leading cause of cancer mortality worldwide. A variety of factors, including genetic alterations and tumor environment, contribute to lung cancer development and drug resistance. High interleukin-6 (IL-6) expression is common in patients with lung cancer and is indicative of poor prognosis^1,2^. IL-6 is released from tumor cells and immune cells and is involved in tumor initiation, progression, and metastasis via activation of multiple intracellular signaling pathways, including the Janus kinase (JAK)/signal transducer and activator of transcription (STAT), phosphoinositide-3 kinase/AKT, RAS/mitogen-activated protein kinase (MAPK), MEK/extracellular signal-regulated kinase 5 (ERK5), and p38/JNK pathways^3–5^. Binding of IL-6 to IL-6R activates the JAK tyrosine kinases to induce phosphorylation of STAT3 at Y705, thereby activating it. This activated STAT3 plays crucial roles in proliferation, survival, metastasis, and self-renewal of cancer cells^6–8^. In lung cancer cells, STAT3 is activated by tyrosine kinases such as epidermal growth factor (EGF) receptor, JAK2, Src, and IL-6R^3,5,9,10^. Phosphorylated STAT3 molecules form homodimers, which enter the nucleus and transcribe target genes, such as survivin, Bcl-2, Mcl-1, c-Myc, cyclin D1, slug, and matrix metalloproteinase-2, involved in tumorigenesis, survival, and metastasis^7,8,11–14^. Aberrant activation of STAT3 is associated with malignant cancers with poor clinical prognosis, and STAT3 inhibition causes cancer cell death, suggesting STAT3 as a potential target for cancer therapy^7,8,15^.

Phosphatases (protein tyrosine phosphatases (PTPs)) control the Y705 phosphorylation-mediated activation of STAT3. PTPs are composed of approximately 280 amino acids with a conserved HCX_5_R motif^16,17^. These enzymes are divided into classical PTPs and dual-specificity phosphatases (DSPs). The classical PTPs include 21 receptor-type PTPs (RPTPs) or 16 non-transmembrane proteins (NT-PTPs). The intracellular region of RPTPs contains the PTP domain with phosphatase activity, whereas the extracellular region shows functions similar to those of cell adhesion molecules and is involved in cell–cell and cell–matrix contact^16^. The NT-PTPs contain catalytic domains with phosphatase activity and non-catalytic domains that regulate subcellular distribution by restricting access to their particular substrates^16^. STAT3 inactivation occurs via dephosphorylation by PTPs such as SH2-containing phosphatase 2 (SHP2), T cell PTP (TC-PTP), and low molecular weight-DSP2^18–20^. Recent studies reported that STAT3 is dephosphorylated by RPTPs, including PTPRT, PTPRD, and PTPRK in colorectal cancer, glioblastoma multiforme, and nasal-type natural killer/T cell lymphoma, respectively^21–23^. PTPRT and PTPRD are tumor suppressors and are frequently inactivated or mutated in human cancers^23,24^.

In addition to PTPs, various negative regulators of STAT3 signaling in cancer cells are known. Protein inhibitor of activated STAT (PIAS1) and suppressor of cytokine-signaling proteins 3 (SOCS3) negatively regulate STAT3 in cancer. PIAS1 can suppress JAK-STAT3 signaling and bind to activated STAT3 dimers to prevent DNA binding for transcription^25,26^. SOCS3 also negatively regulates JAK-STAT3, p44/P42 MAPK, and p53 in prostate and hepatocellular cancer^27–29^. STAT3-interacting proteins, including chromatin remodeling proteins such as BRG1 and oncogenic transcription factors such as nuclear factor kB (NF-kB) or nuclear factor of activated T cells 1 (NFATc1), have been reported to function as either positive or negative regulators in many types of cancers^30^. Of the positive regulators, annexin A2 binds to STAT3 C-terminal domain containing transactivation domain (TAD) and enhances STAT3 activity, thereby promoting epithelial-to-mesenchymal transition in breast cancer^31^. In contrast, GdX (X-linked gene in G6PD cluster at Xq28) is a negative regulator of STAT3 and promotes STAT3 dephosphorylation by stabilizing the interaction between STAT3 and TC45 (the nuclear isoform of TC-PTP), a nuclear phosphatase of STAT3^32^.

DNA damage-induced apoptosis suppressor (DDIAS), which is abundantly expressed in lung cancer and hepatocellular carcinoma (HCC), regulates the survival of cancer cells via multiple signaling pathways^33–36^. DDIAS expression is activated by the transcription factor NFATc1 and is induced by ERK5/myocyte enhancer factor 2B pathway^33,34^. Moreover, DDIAS protein stability is negatively regulated by the E3 U-box ubiquitin ligase carboxyl terminus of heat shock protein 70-interacting protein^35^. Furthermore, DDIAS acts as a cofactor for DNA polymerase–primase complex by associating with DNA polymerase to promote tumorigenesis in HCC^37^. DDIAS protects cancer cells from DNA-damaging reagents and tumor necrosis factor-related apoptosis-inducing ligand (TRAIL) and positively regulates cancer cell invasion by stabilizing β-catenin^33,34,36,38^. Hence, DDIAS is suggested as a potential therapeutic target for malignant lung cancer^33,35,36^. Although IL-6 is implicated in the malignancy of lung cancers, the association of DDIAS in IL-6–mediated signaling has not been investigated. Therefore, using DDIAS-knockdown experiments, we investigated the mechanisms underlying the role of DDIAS in the IL-6–mediated pathway.

In this study, we demonstrated that DDIAS positively regulated IL-6–mediated STAT3 activation. We also showed that DDIAS physically associated with STAT3 to prevent recruitment of STAT3 to protein tyrosine phosphatase receptor mu (PTPRM), a novel phosphatase for STAT3, thereby sustaining STAT3 Y705 phosphorylation. Our findings suggest a novel pathway for DDIAS-mediated aberrant activation of STAT3 in malignant lung cancer cells.

## Results

### DDIAS promotes STAT3 tyrosine phosphorylation in the presence of IL-6

**Fig. 1:**
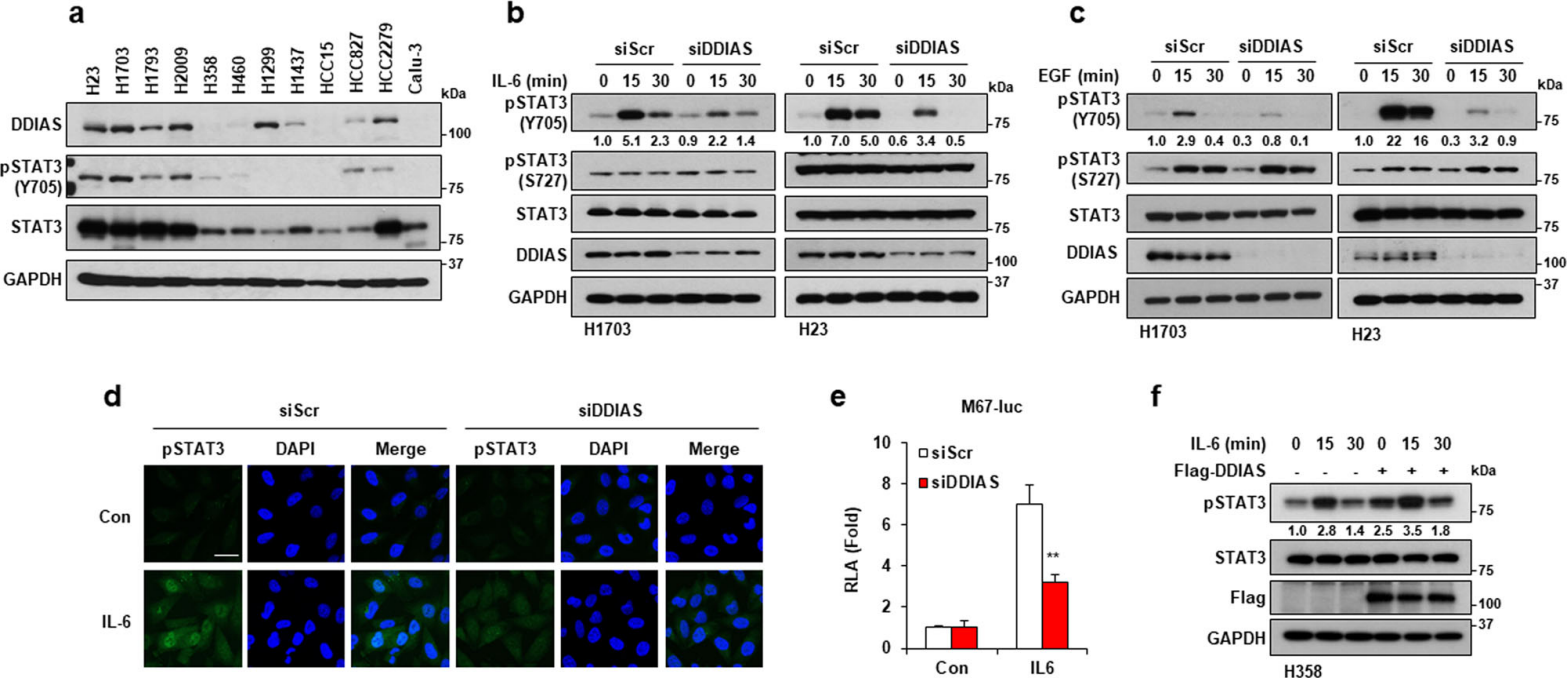
DDIAS depletion suppresses STAT3 activation induced by IL-6 and EGF. **a** Expression of DDIAS and phosphorylated STAT3 in several NSCLC cell lines. **b**, **c** Loss of DDIAS selectively suppressed the STAT3 Y705 phosphorylation in NCI-H1703 and NCI-H23 cells after IL-6 (20 ng/ml) and EGF (100 ng/ml) treatment for the indicated times. **d** Immunocytochemistry for pSTAT3 (Y705) in DDIAS-depleted cells treated with IL-6. Scale bars represent 10 μm. **e** Transactivation of STAT3 by IL-6. NCI-H1703 cells were transfected with siDDIAS and M67-Luc, treated with IL-6 for 6 h, and the relative luciferase activity was determined. The results shown are representative of three experiments (*n* = 3). The values represent means ± SEM. **p < 0.01. **f** Overexpression of DDIAS enhanced the STAT3 Y705 phosphorylation in NCI-H358 cells after IL-6 (20 ng/ml) treatment for the indicated times.

To determine whether IL-6–mediated signaling is controlled by DDIAS, we performed human phospho-kinase array using Proteome Profiler^TM^ antibody array in NCI-H1703 cells. Decreased pSTAT3 (Y705) levels were observed in DDIAS-depleted cells in the presence of IL-6 (Fig. S1). Then we examined the correlation between DDIAS and STAT3 activation in several lung cancer cells. DDIAS expression was strongly correlated with STAT3 expression and STAT3 Y705 phosphorylation (Fig. 1a). To assess whether DDIAS is involved in the IL-6–mediated STAT3 activation, we compared STAT3 phosphorylation in the presence or absence of DDIAS. DDIAS depletion attenuated IL-6–induced STAT3 phosphorylation at Y705 but not at S727 in non-small cell lung cancer (NSCLC) cells, such as NCI-H23 and NCI-H1703 (Fig. 1b). Furthermore, DDIAS knockdown suppressed EGF-induced STAT3 Y705 phosphorylation (Fig. 1c) and inhibited the nuclear translocation of IL-6–mediated pSTAT3 (Fig. 1d). To address whether DDIAS regulates STAT3 transcriptional activity, we performed luciferase assay using a reporter gene (M67-Luc) containing STAT3-binding sites, in the presence or absence of DDIAS. DDIAS depletion significantly repressed the IL-6–induced STAT3 Y705 transcriptional activity in NCI-H1703 (Fig. 1e), whereas Flag-DDIAS overexpression enhanced IL-6–induced STAT3 phosphorylation (Fig. 1f). These results suggest that DDIAS is involved in enhancing STAT3 phosphorylation in the presence of IL-6 or EGF.

### PTP inhibition overcomes STAT3 dephosphorylation in DDIAS-knockdown cells

**Fig. 2:**
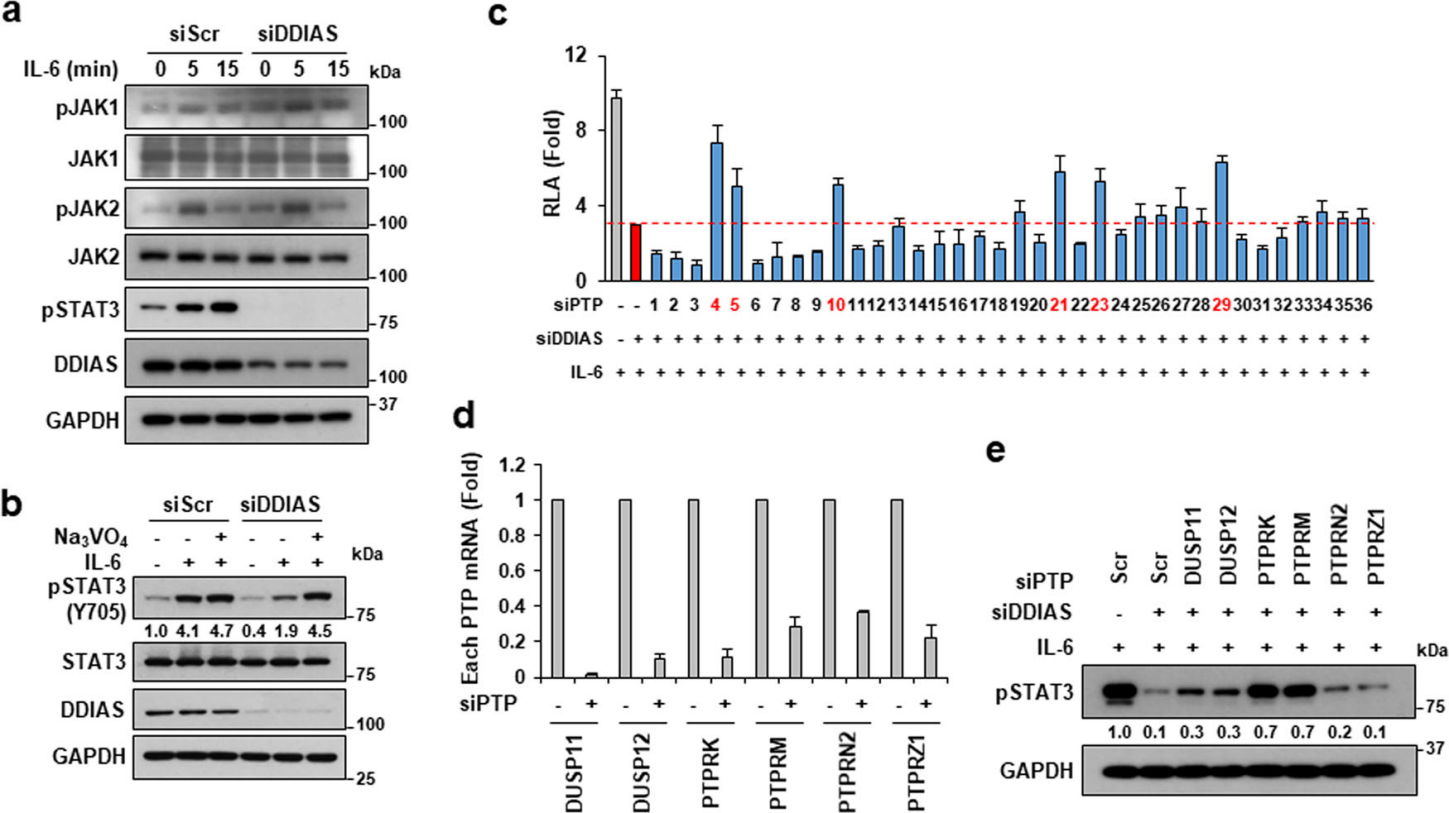
Identification of a phosphatase for STAT3 in DDIAS-depleted cells. **a** DDIAS knockdown did not affect the JAK pathway. NCI-H1703 cells were treated with IL-6 (20 ng/ml) for the indicated times, and western blotting was performed with the indicated antibodies. **b** Protein tyrosine phosphatase (PTP) inhibition recovered the DDIAS-knockdown-mediated dephosphorylation of STAT3. NCI-H1703 cells transfected with siDDIAS were pretreated with 1 mM sodium vanadate for 4 h and treated with IL-6 for 15 min. **c** siRNA-based screening for identification of PTPs involved in STAT3 dephosphorylation in the absence of DDIAS. The levels of STAT3 transcriptional activity were measured by a reporter assay (*n* = 3). **d** Relative mRNA level of each PTP gene. RT-qPCR was performed 72 h after siRNA transfection. All experiments were independently performed three times, and each experiment was performed in triplicate. **e** Knockdown of PTPRK or PTPRM recovered the DDIAS-knockdown-induced decrease in STAT3 phosphorylation.

To understand the mechanism underlying STAT3 activation by DDIAS in the presence of IL-6, we investigated the phosphorylation status of JAK1 and JAK2. No significant change was detected in either expression level or phosphorylation degree of both JAK1 and JAK2 in DDIAS-silenced cells (Fig. 2a). Thus the JAK pathway may not be associated with the decrease in STAT3 phosphorylation in the DDIAS-knockdown cells. Therefore, to assess whether DDIAS is involved in the regulation of PTP-mediated dephosphorylation of STAT3, we treated DDIAS-depleted cells with sodium vanadate, a PTP inhibitor, in the presence of IL-6. Sodium vanadate completely recovered STAT3 dephosphorylation in DDIAS-knockdown NCI-H1703 cells (Fig. 2b). Next, we explored the PTPs involved in STAT3 dephosphorylation in the absence of DDIAS. Small interfering RNA (siRNA) screening of validated siRNAs of 36 PTPs was performed to identify PTPs that could recover STAT3 Y705 phosphorylation. Six candidates PTPRM, PTPRN2, DUSP11, PTPRK, PTPRZ1, and DUSP12 overcame the suppression of STAT3 phosphorylation and its transcriptional activity in DDIAS-knockdown cells (Fig. 2c, Fig. S2). The knockdown efficiency of siRNA against the six genes was confirmed using quantitative real-time polymerase chain reaction (qPCR) (Fig. 2d). PTPRK knockdown and PTPRM knockdown completely recovered the DDIAS-knockdown-induced decrease in STAT3 phosphorylation (Fig. 2e). These results demonstrate that DDIAS is involved in the activation of STAT3 signaling by suppressing that activity of PTPs, such as PTPRM or PTPRK.

### PTPRM is a novel phosphatase of STAT3

**Fig. 3:**
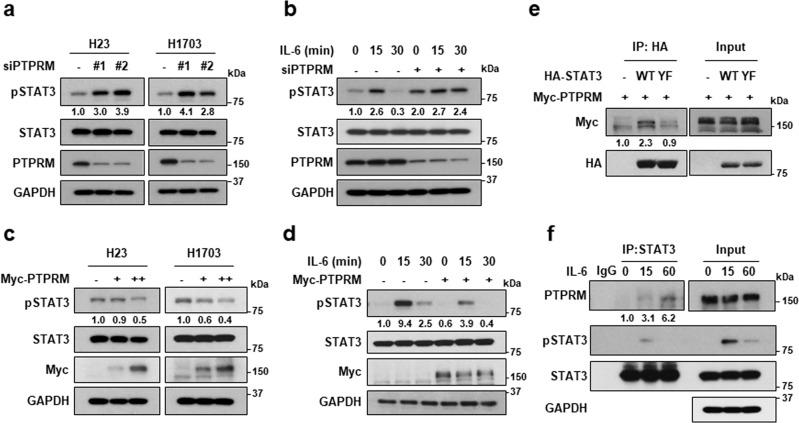
PTPRM dephosphorylates STAT3 by direct interaction. **a**, **b** PTPRM knockdown increased STAT3 phosphorylation in NCI-H23 and NCI-H1703 cells. Cells were transfected with siPTPRM (#1, #2) for 72 h (**a**) and treated with IL-6 (**b**). Western blotting was performed. **c**, **d** PTPRM overexpression inhibited STAT3 phosphorylation. Cells were transfected with Myc-PTPRM for 48 h (**c**) and treated with IL-6 (**d**). Western blotting was performed. **e** Interaction between Myc-PTPRM and HA-STAT3 WT or YF (Y705F). Immunoprecipitation was performed using anti-HA antibody. **f** Interaction between PTPRM and STAT3 in the presence of IL-6. Immunoprecipitation was performed using anti-STAT3 antibody.

To confirm that PTPRM regulates STAT3 Y705 dephosphorylation, PTPRM siRNA was transfected into NCI-H23 and NCI-H1703 cells. Compared to the respective controls, PTPRM knockdown cells showed an increased basal level of STAT3 phosphorylation and increased levels of IL-6–induced STAT3 phosphorylation (Fig. 3a, b), whereas cells with Myc-PTPRM overexpression showed decreased levels of both basal and IL-6–induced STAT3 phosphorylation (Fig. 3c, d). Hence, we next assessed the importance of Y705 for the interaction between PTPRM and STAT3. Co-immunoprecipitation assay revealed that Myc-PTPRM bound to hemagglutinin (HA)-STAT3 wild type (WT) but not HA-STAT3 Y705F (YF; Fig. 3e), indicating that PTPRM is associated with Y705 dephosphorylation of STAT3. Interestingly, PTPRM and STAT3 showed a strong interaction at 60 min of IL-6 treatment (Fig. 3f). These results indicate that PTPRM is a novel phosphatase for STAT3.

### DDIAS inhibits PTPRM-STAT3 binding to sustain STAT3 phosphorylation

**Fig. 4:**
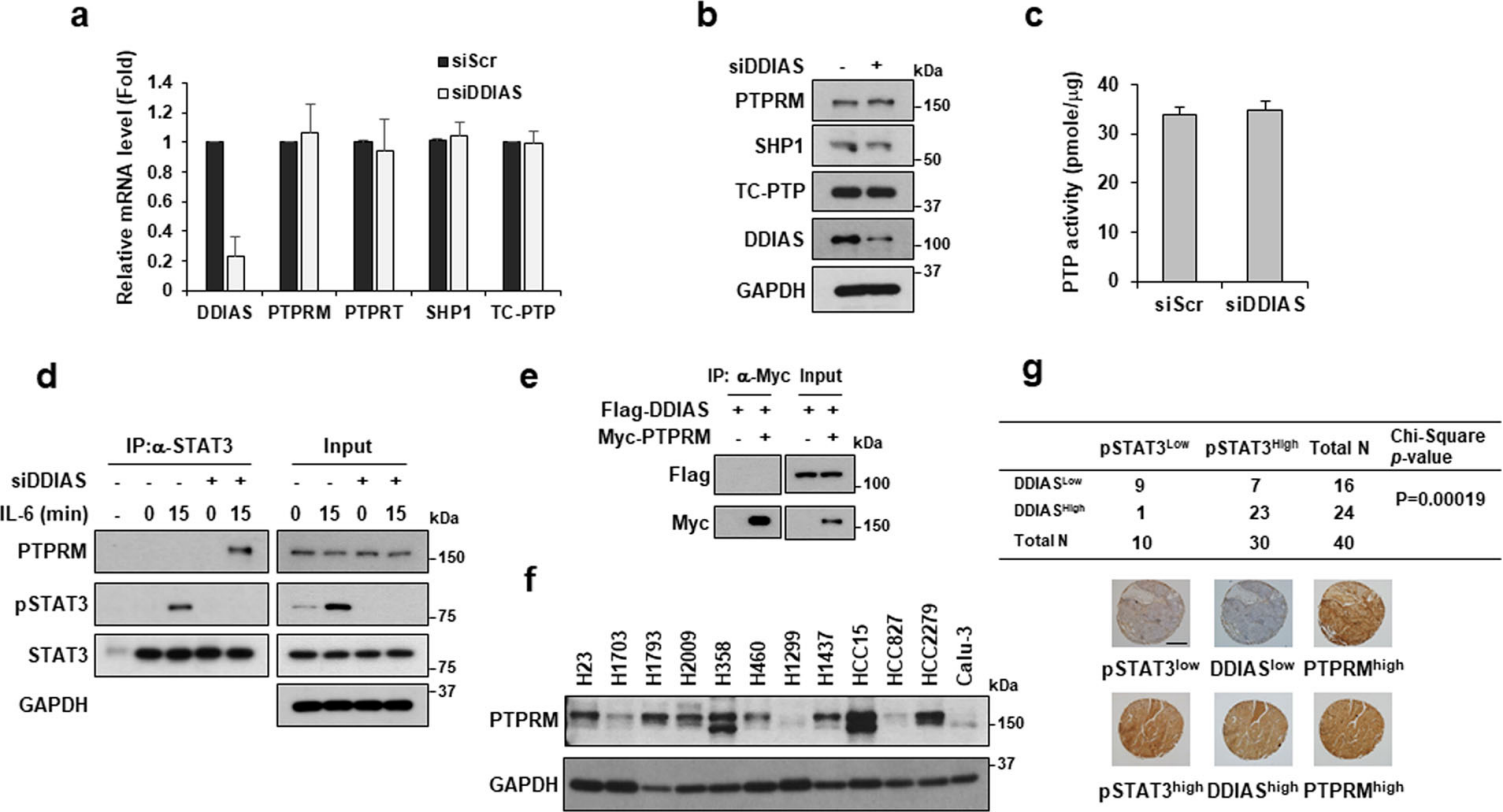
DDIAS inhibits the interaction between STAT3 and PTPRM. **a** mRNA levels of PTPRM, PTPRT, SHP1, and TC-PTP in DDIAS-knockdown cells. RT-qPCR was performed 72 h after siDDIAS transfection. All experiments were independently performed three times, and each experiment was performed in triplicate. **b** Western blot analysis of PTPRM, SHP1, and TC-PTP in DDIAS-knockdown cells. **c** Activity of total PTP in DDIAS-knockdown cells. **d** Interaction between PTPRM and STAT3 in the presence of IL-6 in DDIAS-knockdown cells. Immunoprecipitation was performed using anti-STAT3 antibody. **e** Interaction between Myc-PTPRM and Flag-DDIAS. Immunoprecipitation was performed using anti-Myc antibody. **f** PTPRM expression in different NSCLC cell lines. **g** DDIAS, PTPRM, and pSTAT3 (Y705) expression in a human lung cancer tissue array. Representative images of LC tissue stained with an anti-DDIAS (1:100), anti-PTPRM (1:200), and anti-pSTAT3 antibodies (1:100). Scale bar, 500 μm.

To understand the association of DDIAS in STAT3 phosphorylation, we investigated the effect of DDIAS on PTPRM activity. First, we examined whether DDIAS depletion affects the mRNA and protein levels of PTPRM. Reverse transcription (RT)-qPCR and western blot analysis revealed that DDIAS did not affect the expression of PTPRM as well as the known STAT3 phosphatases PTPRT, SHP1, and TC-PTP (Fig. 4a, b). Next, we determined the effect of DDIAS on PTP activity using in vitro phosphatase activity assay in DDIAS-knockdown cells. Consistently, DDIAS knockdown did not affect the total PTP activity (Fig. 4c). Therefore, we performed immunoprecipitation analysis using anti-STAT3 antibody in DDIAS-knockdown cells to investigate whether DDIAS is involved in the regulation of PTPRM/STAT3 binding. Notably, in the presence of IL-6, PTPRM and STAT3 showed a stronger interaction in DDIAS-knockdown cells, compared with that in the control cells (Fig. 4d). Next, we performed co-immunoprecipitation analysis to determine whether DDIAS interacts with PTPRM and found that Flag-DDIAS did not interact with Myc-PTPRM in HEK293T cells (Fig. 4e). This result suggests that DDIAS interferes with the interaction between PTPRM and STAT3 without binding to PTPRM. Considering these observations, we investigated the correlation between the expression of PTPRM, STAT3, and DDIAS in different NSCLC cell lines. Interestingly, PTPRM expression in the different NSCLC cell lines was variable and showed no correlation with DDIAS or STAT3 expression in most of the cell lines (Figs. 4f and 1a). To evaluate the correlation between STAT3 activation and expression of DDIAS and PTPRM, we performed immunohistochemistry (IHC) on 40 human lung cancer tissues (Fig. 4g). Consistent with our in vitro human lung cancer cell lines (Fig. 1a), DDIAS expression level was strongly correlated with STAT3 tyrosine phosphorylation. However, overall PTPRM expression level has no correlation with STAT3 phosphorylation in human lung cancer tissues.

### DDIAS interacts with STAT3 and interferes with PTPRM/STAT3 binding

**Fig. 5:**
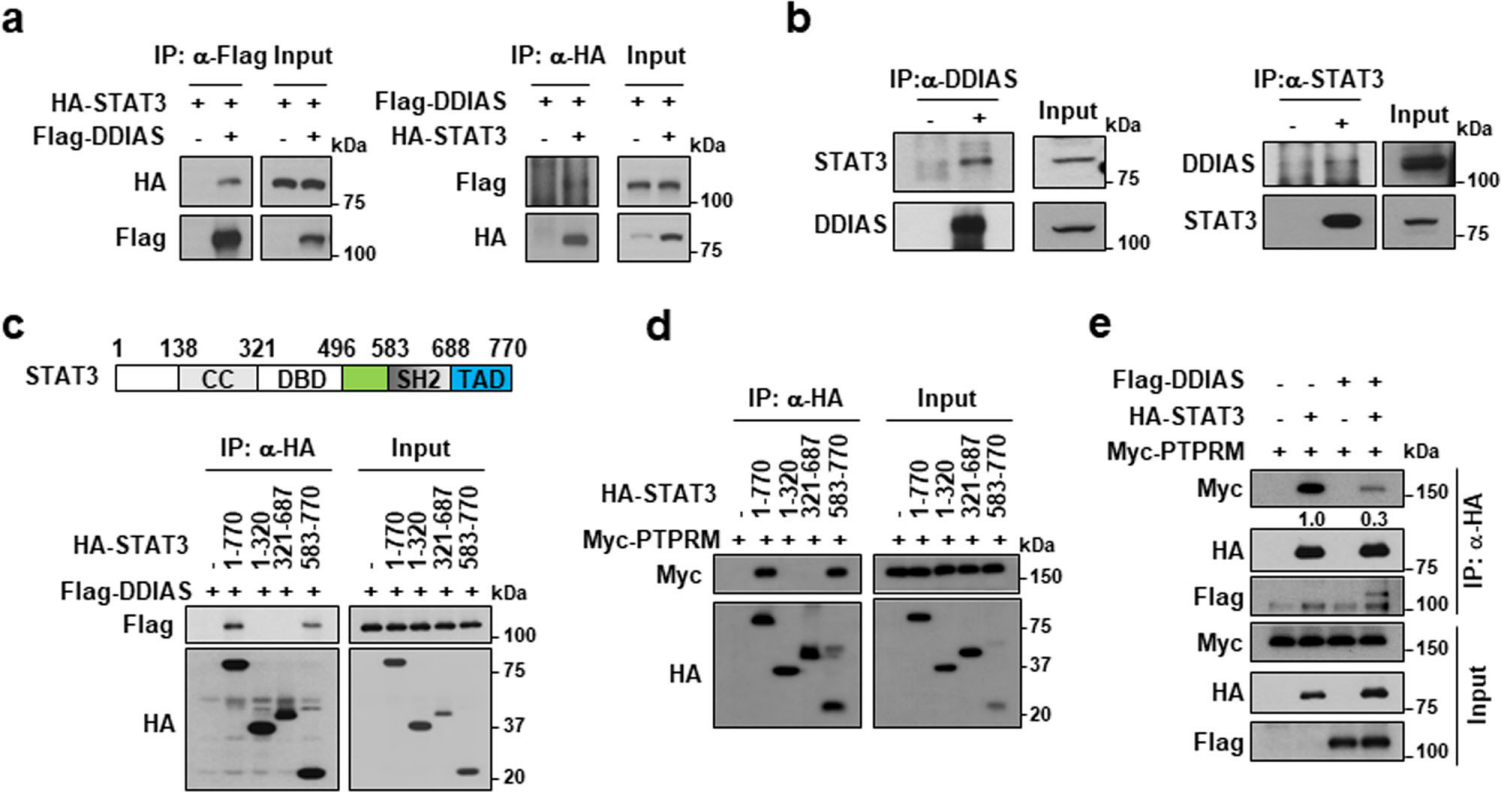
DDIAS associates with STAT3. **a** DDIAS binds to STAT3. Flag-DDIAS and HA-STAT3 were co-transfected into HEK293T cells in the indicated combinations. The cell lysates were subjected to an immunoprecipitation assay using anti-Flag agarose or anti-HA beads, and the immunoprecipitates were probed with anti-HA or anti-Flag antibody. **b** Binding of endogenous STAT3 and DDIAS. Immunoprecipitation was performed using anti-STAT3 or anti-DDIAS in NCI-H1703 cells. **c** Mapping of STAT3-binding region on DDIAS. Flag-DDIAS and HA-STAT3 deletion constructs were co-transfected into HEK293T cells in the indicated combinations, and their interactions were analyzed by co-immunoprecipitation. **d** Mapping of STAT3-binding region on PTPRM. HA-STAT3 deletion constructs and Myc-PTPRM were co-transfected into HEK293T cells in the indicated combinations. The cell lysates were subjected to an immunoprecipitation assay using anti-HA antibody, and the immunoprecipitates were probed with anti-Myc or anti-HA antibody. **e** Effect of Flag-DDIAS on the interaction between STAT3 and PTPRM. HEK293T cells co-transfected with FLAG-DDIAS and/or Myc-PTPRM and HA-STAT3. Co-immunoprecipitation was performed using anti-HA antibody.

To further understand the mechanism underlying DDIAS-mediated regulation of STAT3 phosphorylation, we assessed whether DDIAS directly interacts with STAT3. Co-immunoprecipitation assay clearly showed that Flag-DDIAS interacted with HA-STAT3 in HEK293T cells (Fig. 5a). Furthermore, endogenous DDIAS bound to STAT3 in NCI-H1703 cells (Fig. 5b). Domain mapping analysis revealed that DDIAS bound to the TAD of STAT3 (Fig. 5c) and that this STAT3 domain was also involved in the interaction with PTPRM (Fig. 5d). Notably, DDIAS overexpression suppressed the interaction between STAT3 and PTPRM (Fig. 5e). These results suggest that DDIAS binds to STAT3 to interfere with PTPRM/STAT3 binding and that PTPRM competes with DDIAS to recruit STAT3 for dephosphorylation.

### DDIAS promotes STAT3-mediated malignancy by preventing PTPRM function

**Fig. 6:**
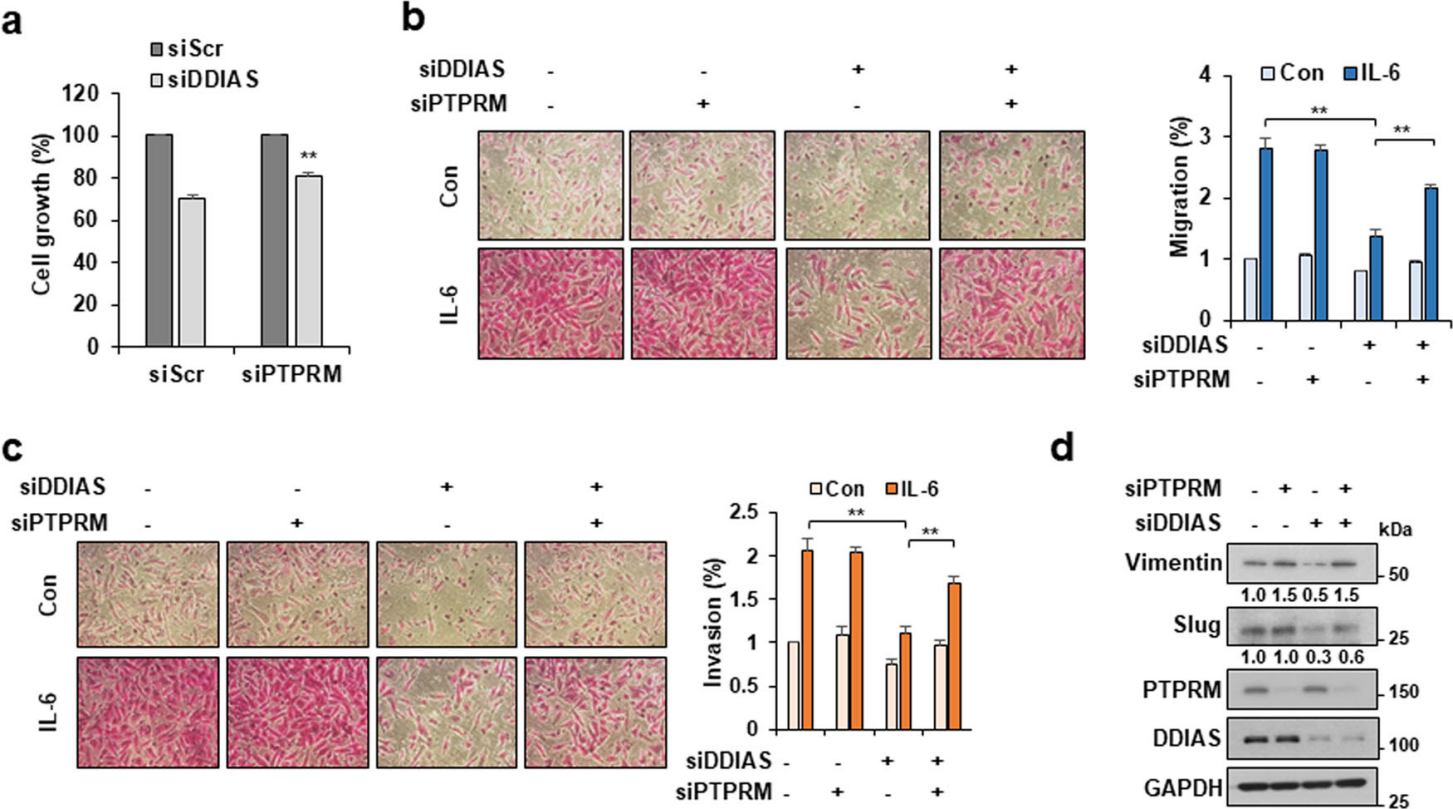
PTPRM inhibits DDIAS-mediated STAT3 signaling. **a** Cell growth assay was performed at 72 h after DDIAS siRNA or/and PTPRM siRNA transfection. The values represent mean ± SEM from three independent experiments with biological triplicate. **p < 0.01. **b**, **c** Migration (**b**) and invasion (**c**) assay were performed 12 or 24 h following IL-6 treatment in cells transfected with DDIAS siRNA or/and PTPRM siRNA. The results shown are representative of three experiments (*n* = 3). The values represent mean ± SEM. **p < 0.01. **d** Levels of STAT3 targets. Western blotting analysis was performed at 24 h after IL-6 treatment in cells transfected with DDIAS siRNA or/and PTPRM siRNA.

We observed that PTPRM overexpression inhibited cell growth in a dose-dependent manner (Fig. S3). Then we examined the effect of PTPRM knockdown on DDIAS-knockdown–induced suppression of cancer cell growth and invasion (Fig. 6). Clearly, PTPRM knockdown partially rescued the cell growth inhibition induced by DDIAS knockdown (Fig. 6a). Moreover, PTPRM silencing recovered the migration and invasion suppressed by DDIAS knockdown (Fig. 6b, c). As expected, PTPRM depletion increased the expression of STAT3 targets vimentin and slug, which are involved in the migration of DDIAS-knockdown cells (Fig. 6d). Thus PTPRM suppresses STAT3 signaling in DDIAS-knockdown cells, suggesting that DDIAS promotes the IL-6–mediated STAT3 signaling in malignant cancer cells by inhibiting the PTPRM function.

## Discussion

IL-6 and STAT3 are implicated in the malignancy of lung cancers, and many studies have attempted to target IL-6/IL-6 receptor for drug development^4^. STAT3 activity is regulated via posttranslational modifications, such as phosphorylation, acetylation, oxidation, and ubiquitination^39–42^. Of these, IL-6–mediated phosphorylation mainly controls STAT3 activity, which is reversibly regulated by protein tyrosine kinases or PTPs^19,20,43^. The known STAT3-binding phosphatases are receptor-type PTPs such as PTPRK, PTPRD, and PTPRK and non-receptor PTPs, including SHP1, SHP2, PTP-MEG2, and TC-PTP. These PTPs are frequently mutated or inactivated in lung cancer, colorectal cancer, glioblastoma, head and neck squamous cell carcinomas, and renal cell carcinoma^21,23,24,44–46^, suggesting that their inactivation contributes to the malignancy of human cancers.

In this study, we found that DDIAS positively regulated IL-6–mediated STAT3 activation. Using siRNA PTP library screening, we first identified PTPRM as a novel PTP that suppresses STAT3 Y705 phosphorylation in NCI-H1703 cells. Furthermore, we found that PTPRK or PTPRT also recovered the STAT3 dephosphorylation induced by DDIAS knockdown (Fig. 2e, Fig. S4). In DDIAS-knockdown cells, PTPRM knockdown recovered the decrease in IL-6–mediated STAT3 Y705 phosphorylation. Similar to PTPRK, PTPRT, and PTPRU, PTPRM belongs to R2B subfamily of RPTPs, which consist of extracellular regions containing one MAM (meprin/A5/μ), one immunoglobulin (Ig)-like, and four fibronectin domains and intracellular regions containing the juxtamembrane domain and tandem intracellular phosphatase domain^17^. PTPRM facilitates hemophilic cell–cell interaction through the extracellular region independent of phosphatase activity in the intracellular domains^47^. Indeed, PTPRM has been shown to inhibit cancer cell proliferation, migration, and invasion^48–50^. PTPRM expression is downregulated by genetic and epigenetic alterations such as loss of heterozygosity and hypermethylation in breast cancer, colorectal cancer, and acute lymphoblastic leukemia^48,49,51^. Moreover, PTPRM is downregulated by miRNA-221/-222 in glioblastoma, suggesting its tumor-suppressor function^52^.

**Fig. 7:**
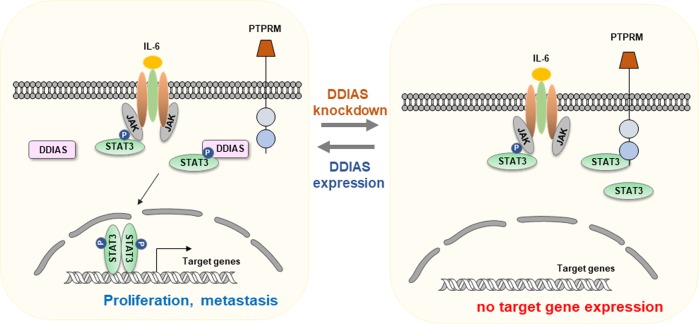
Model showing DDIAS function in STAT3 regulation. DDIAS expression contributes to constitutive STAT3 tyrosine phosphorylation via inhibition of protein tyrosine phosphatases in lung cancers.

Mechanistically, non-receptor PTPs, such as SHP1or SHP2 containing Src homology-2 (SH2) domains, mediate the interaction of PTP with its substrates through SH domains, which function as phospho-tyrosine-binding domains. While receptor-type PTPs such as PTPRK and PTPRT have two intracellular PTP domains (D1 and D2), mutants of the D1 and D2 domain show decreased PTP activity^21,22,44^. However, it is believed that the D2 domain of RPTP is catalytically inactive and functions as a substrate-binding domain that modifies the RPTP structure^53^. It has been shown that PTPRK directly binds to STAT3 and dephosphorylates STAT3 Y705^1^. In contrast, PTPRM dephosphorylates catenin p120^ctn^ by binding to the N-terminus of catenin p120^ctn^, which is important for tyrosine phosphorylation^54–56^. Our data show that PTPRM interacted with the STAT3 TAD, which contains the Y705 (Fig. 7). We also observed that DDIAS bound to the STAT3 C-terminal domain containing TAD. These findings suggest that DDIAS competes with PTPRM for STAT3 binding. Thus DDIAS sustains STAT3 phosphorylation by inhibiting the accessibility of PTPRM to STAT3, suggesting a novel mechanism of PTPRM as a tumor suppressor.

We found that DDIAS expression is closely correlated with STAT3 Y705 phosphorylation in NSCLC cell lines and that PTPRM knockdown recovered the reduced STAT3 Y705 phosphorylation in DDIAS-knockdown cells. However, PTPRM expression was variable and showed no correlation with STAT Y705 phosphorylation in lung cancer cell lines and human tissues (Fig. 4f, g); this suggests that mutations in PTPRM or other PTPs are involved in STAT3 dephosphorylation, depending on the cancer cells. The protein levels of PTPRK in the different NSCLC cell lines were also variable and showed no correlation with STAT3 Y705 phosphorylation (Fig. S5a). In NCI-H1703 cells, the expression level of PTPRM was higher than that of PTPRK, PTPRT, or PTPRZ1 in NCI-H1703 cells (Fig. S5b), suggesting that PTPRM is the main phosphatase for STAT3. However, it may be possible that DDIAS prevents STAT3 from recruiting other RPTPs, including PTPRK or PTPRT, by directly binding with STAT3 in other cells. PTPRT expression was very low in most of the NSCLC cell lines (Fig. S5a). Notably, DDIAS affects the involvement of phosphatases in regulation of IL-6–mediated STAT3 phosphorylation.

Previously, we showed that DDIAS promotes proliferation and invasion of cancer cells and is involved in suppression of DNA-damaging-induced apoptosis and TRAIL-mediated extrinsic apoptosis in cancer cells. In this study, we demonstrated that DDIAS promoted IL-6–mediated STAT3 activation in lung cancer cells by competing with PTPRM. DDIAS knockdown caused growth inhibition of lung cancer cells through STAT3 inactivation by PTPRM, suggesting DDIAS as a potential therapeutic target in aberrant STAT3-activated lung cancer. Moreover, IHC analysis demonstrated correlation between the DDIAS expression level and STAT3 phosphorylation in lung cancer patient tissues (Fig. S6). Therefore, we suggest that DDIAS can serve as a biomarker to predict the STAT3 phosphorylation in malignant lung cancer.

We newly identified the DDIAS/PTPRM/STAT3 system in lung cancer cells with aberrant STAT3 expression. We expect multiple, intertwined crosstalks and interactions between DDIAS/PTPRM and other STAT3 regulation system in lung cancer cells. However, we do not have conclusive evidence regarding it. The ability of PTP to compete with DDIAS for STAT3 inactivation depends on its specificity to STAT3, its structural similarity to PTPRM, and its intracellular availability. PTPRM belongs to the same family as PTPRK and PTPRT. Although PTPRT has specificity to STAT3, it is less abundant than PTPRM in the cell. Non-receptor-type PTP, such as SHP1, SHP2, and TC-PTP, are not specific to STAT3. Even though TC-PTP is present at a high level, the dephosphorylation efficiency of STAT3 by TC-PTP is expected to be lower than that by PTPRM. Interestingly, DUSP11 and DUSP12 seem to affect STAT3 inactivation through MAP inhibition.

Using siRNA PTP library screening, we identified PTPRM as an activator of STAT3 Y705 phosphorylation in DDIAS-knockdown cells. However, the PTP siRNA library does not contain all the known PTPs; therefore, it is possible that we may have missed a PTP involved in STAT3 Y705 dephosphorylation. Moreover, a PTP could have been excluded as a false negative in our siRNA screening system, although the experiment using validated siRNA was performed under stringent conditions. Nevertheless, we demonstrated that DDIAS binds to STAT3 and prevents STAT3 recruitment to PTPRM for Y705 dephosphorylation. We further plan to screen drugs inhibiting the interaction between DDIAS and STAT3 and investigate the effectiveness of this drug in combination with the currently available treatment for lung cancer.

In summary, we provide novel insights into the crucial role of DDIAS in STAT3 signaling in malignant lung cancer. We confirmed that DDIAS is involved in STAT3 activation in malignant lung cancer. We report that PTPRM is a novel STAT3 tyrosine phosphatase and that it negatively regulates STAT3 activation by dephosphorylating Y705 of STAT3 in the presence of IL-6. We also demonstrate that DDIAS promotes STAT3 phosphorylation by blocking PTPRM recruitment of STAT3 via competitive binding with STAT3.

## Materials and methods

### Reagents, antibodies, and plasmids

EGF, IL-6, and human phospho-kinase array were purchased from R&D Systems (Minneapolis, MN, USA). Other chemicals were purchased from Sigma-Aldrich (St. Louis, MO, USA). The following antibodies were used: anti-DDIAS (HPA038540) from Atlas Antibodies (Stockholm, Sweden); anti-SHP1 (sc-7289), anti-TC-PTP (sc-373835), anti-HA (sc-805), and anti-Myc (sc-40) from Santa Cruz Biotechnology (Santa Cruz, CA, USA); anti-STAT3 (9139, 4904), anti-HA (3724), anti-pSTAT3 (Y705, 9145), anti-pSTAT3 (S727, 9134), anti-pJAK1 (Y1034/1035, 3331), anti-JAK1 (3344), anti-pJAK2 (Y1007/1008, 3771), and anti-JAK2 (3230) from Cell Signaling Technology (Beverly, MA, USA); anti-PTPRM (MAB4446) from R&D Systems; anti-GAPDH (LF-P-A0212) from AbFrontier (Seoul, Korea); and anti-Flag (F1804) from Sigma-Aldrich. Full-length and deletion mutants of Flag-DDIAS were cloned as previously described^35^. 4×M67 pTATA TK-Luc (#8688) and Flag-STAT3 Y705F (#24984) were obtained from Addgene (Cambridge, MA, USA). Myc-DDK-tagged PTPRM (RC226398) was obtained from OriGene (Rockville, MD, USA). Full-length and deletion mutants of STAT3-tagged HA were subcloned from the Flag-STAT3 expression vector (Addgene). STAT3 gene was mutated using the QuickChange Site-Directed Mutagenesis Kit (Enzynomics, Daejeon, Korea). The constructs were verified using sequence analysis.

### Cell culture, transfections, and cell viability

Human embryonic kidney 293T (HEK293T), Calu-3, and human NSCLC cell lines NCI-H23, NCI-H1703, NCI-H1793, NCI-H2009, NCI-H358, NCI-H460, NCI-H1299, NCI-H1437, HCC15, HCC827, and HCC2279 were purchased from Korean Cell Line Bank (Seoul, Korea) or KRIBB Cell Line Bank (Daejeon, Korea). Calu-3 and HEK293T cells were cultured in Dulbecco’s modified Eagle’s medium and all NSCLC cell lines in RPMI-1640 supplemented with 10% fetal bovine serum and penicillin/streptomycin (Invitrogen, Carlsbad, CA, USA). Cells were transfected with the indicated plasmids using TurboFect (Thermo Scientific, Rockford, IL, USA) according to the manufacturer’s instructions and siRNAs (20–40 nM) by electroporation (Neon, Invitrogen) according to the manufacturer’s instructions. Cell viability was determined using the sulforhodamine B (SRB) assay, as previously described^57^.

### Small-interfering RNA

*AccuTarget™* Genome-wide validated siRNA Library and siRNAs used in this study were purchased from Bioneer Corporation (Daejeon, Korea). The target sequences were as follows: siDDIAS: 5′- CAGAAGAGAUCUGCAUGUU-3′, siDUSP11: 5′-CUAUUCACACAGGAGGUAU-3′, siDUSP12: 5′-CAUUCAUGGCAGAUUGUUU-3′, siPTPRM (#1): 5′-CGAGCUAUAAAAUUGGACA-3′, siPTPRM (#2): 5′-CUGGUUACAGGGCAUUGAU-3′, siPTPRK: 5′-GCCCAGACUAAGAACAUCAAU-3′, siPTPRN2: 5′-AGUAUCCGAUUCGCCAUCA-3′, siPTPRZ1: 5′-GACAUGGGAGUACCAGAGU-3′, or Scrambled: 5′-CCUACGCCACCAAUUUCGU-3′.

### Luciferase reporter assays

Cells were co-transfected with 300 ng of 4×M67 pTATA TK-Luc and 10 ng of pRL-TK using 1 μl of TurboFect per well in 24-well plates, serum starved for 24 h, and treated with 20 ng/ml of IL-6 for 6 h. Luciferase assay was performed as previously described^33^.

### Quantitative PCR

Total RNA isolation, RT, and qPCR were performed as described previously^34^. The primers for DUSP11 (P318718), DUSP12 (P266589), PTPRK (P109949), PTPRM (P153012), PTPRN2 (P103059), PTPRZ1 (P254869), PTPRT (P315908), SHP1 (P277701), TC-PTP (P205396), and STAT3 (P229000) were obtained from Bioneer (Daejeon, Korea). Primers used for DDIAS and glyceraldehyde 3-phosphate dehydrogenase (GAPDH) were as described previously^33^. All reactions were performed in triplicate and normalized to GAPDH as the internal control.

### Cell migration and invasion assay

These assays were performed in 24-well Transwell plates (BD Biosciences, San Jose, CA, USA) with 8-μm pore inserts coated with or without Matrigel (Invitrogen). Cells (1 × 10^5^) were seeded in a culture insert in serum-free media and treated with 20 ng/ml IL-6 into the upper chamber. Complete medium was applied to the lower chamber. Cells were allowed to migrate or invade the layer for 12 or 24 h, respectively. Migrating/invading cells were stained with 0.4% SRB and visualized under a microscope. The experiment was repeated three times.

### Immunocytochemistry

Immunofluorescence staining was performed as described previously^34^. Cells were incubated with an anti-pSTAT3 antibody overnight. Fluorescent images were visualized using an LSM 800 microscope (Zeiss, Jena, Germany).

### Immunohistochemistry

Human tissue arrays (LC485) were obtained from US Biomax, Inc. (Rockville, MD, USA). IHC staining was performed as previously described^33^. Tissues were incubated with anti-pSTAT3 (Y705), anti-DDIAS, or anti-PTPRM antibodies overnight. The images were visualized using an Olympus BX51 microscope equipped with a DP71 digital camera and DP-B software (Olympus Co, Tokyo, Japan). pSTAT3, DDIAS, and PTPRM positivity was defined as 3+, 2+, 1+, or 0 by IHC. Tissues stained were divided into two high (3+ and 2+) and low (1+ and 0) groups. Scoring of the human tissue array was performed by two independent observers (J.-Y.I. and K.-W.L.), showing high consistency between scores for both pSTAT3 and DDIAS.

### Co-immunoprecipitation and western blot analysis

Immunoprecipitation and western blotting were performed as described previously^36^. For endogenous binding, lysates were incubated with anti-STAT3 or anti-DDIAS antibody and subjected to western blot analysis with specific antibodies. Signals were detected using an enhanced chemiluminescence kit (Millipore, Temecula, CA, USA). The densitometry numbers indicated were measured using Image J (NIH).

### Tyrosine phosphatase assay

PTP activity was measured using a Tyrosine Phosphatase Assay System (Promega, Madison, WI, USA) according to the manufacturer’s protocol. NCI-H1703 cells were transfected with siDDIAS for 72 h and lysed with storage buffer. Spin columns were used to remove endogenous free phosphate from the cell lysates. Tyrosine phosphatase activity was measured using the EMax plate reader system (Molecular Devices, San Jose, CA, USA) at 600 nm.

### Statistical analysis

Data were obtained from at least three independent experiments with biological triplicates and presented as mean ± SEM. Statistical analyses were performed using two-tailed Student’s *t* test. A value of *p* < 0.05 was accepted as significant.

## Supplementary information

Supplementary Figure S1. DDIAS knockdown suppresses IL-6-induced STAT3 phosphorylation

Supplementary Figure S2. siRNA screen for protein tyrosine phosphatases (PTPs) on STAT3 dephosphorylation by DDIAS knockdown

Supplementary Figure S3. Overexpression of PTPRM inhibited lung cancer cell growth

Supplementary Figure S4. The effect of PTPRT silencing on STAT3 dephosphorylation by DDIAS knockdown

Supplementary Figure S5. The level of RPTPs in NSCLC

Supplementary Figure S6. Staining of DDIAS, PTPRM, pSTAT3 (Y705) in human lung cancer tissue array

## References

[1] Chang CH (2013). Circulating interleukin-6 level is a prognostic marker for survival in advanced nonsmall cell lung cancer patients treated with chemotherapy. Int. J. Cancer.

[2] Yanagawa H (1995). Serum levels of interleukin 6 in patients with lung cancer. Br. J. Cancer.

[3] Gao SP (2007). Mutations in the EGFR kinase domain mediate STAT3 activation via IL-6 production in human lung adenocarcinomas. J. Clin. Invest..

[4] Yao X (2014). Targeting interleukin-6 in inflammatory autoimmune diseases and cancers. Pharm. Ther..

[5] Yao Z (2010). TGF-beta IL-6 axis mediates selective and adaptive mechanisms of resistance to molecular targeted therapy in lung cancer. Proc. Natl Acad. Sci. USA.

[6] Bromberg JF (1999). Stat3 as an oncogene. Cell.

[7] Yu H, Jove R (2004). The STATs of cancer-new molecular targets come of age. Nat. Rev. Cancer.

[8] Yu H, Pardoll D, Jove R (2009). STATs in cancer inflammation and immunity: a leading role for STAT3. Nat. Rev. Cancer.

[9] Yeh HH, Lai WW, Chen HH, Liu HS, Su WC (2006). Autocrine IL-6-induced Stat3 activation contributes to the pathogenesis of lung adenocarcinoma and malignant pleural effusion. Oncogene.

[10] Harada D, Takigawa N, Kiura K (2014). The role of STAT3 in non-small cell lung cancer. Cancers (Basel).

[11] Bhattacharya S, Ray RM, Johnson LR (2005). STAT3-mediated transcription of Bcl-2, Mcl-1 and c-IAP2 prevents apoptosis in polyamine-depleted cells. Biochem. J..

[12] Gritsko T (2006). Persistent activation of stat3 signaling induces survivin gene expression and confers resistance to apoptosis in human breast cancer cells. Clin. Cancer Res..

[13] Masuda M (2002). The roles of JNK1 and Stat3 in the response of head and neck cancer cell lines to combined treatment with all-trans-retinoic acid and 5-fluorouracil. Jpn J. Cancer Res..

[14] Xie TX (2004). Stat3 activation regulates the expression of matrix metalloproteinase-2 and tumor invasion and metastasis. Oncogene.

[15] Zhang X (2012). Orally bioavailable small-molecule inhibitor of transcription factor Stat3 regresses human breast and lung cancer xenografts. Proc. Natl Acad. Sci. USA.

[16] Tonks NK (2006). Protein tyrosine phosphatases: from genes, to function, to disease. Nat. Rev. Mol. Cell Biol..

[17] Tonks NK (2013). Protein tyrosine phosphatases-from housekeeping enzymes to master regulators of signal transduction. FEBS J..

[18] Sekine Y (2006). Regulation of STAT3-mediated signaling by LMW-DSP2. Oncogene.

[19] Yamamoto T (2002). The nuclear isoform of protein-tyrosine phosphatase TC-PTP regulates interleukin-6-mediated signaling pathway through STAT3 dephosphorylation. Biochem. Biophys. Res. Commun..

[20] Bard-Chapeau EA (2011). Ptpn11/Shp2 acts as a tumor suppressor in hepatocellular carcinogenesis. Cancer Cell.

[21] Zhang X (2007). Identification of STAT3 as a substrate of receptor protein tyrosine phosphatase T. Proc. Natl Acad. Sci. USA.

[22] Chen YW (2015). Receptor-type tyrosine-protein phosphatase kappa directly targets STAT3 activation for tumor suppression in nasal NK/T-cell lymphoma. Blood.

[23] Veeriah S (2009). The tyrosine phosphatase PTPRD is a tumor suppressor that is frequently inactivated and mutated in glioblastoma and other human cancers. Proc. Natl Acad. Sci. USA.

[24] Lui VW (2014). Frequent mutation of receptor protein tyrosine phosphatases provides a mechanism for STAT3 hyperactivation in head and neck cancer. Proc. Natl Acad. Sci. USA.

[25] Chung CD (1997). Specific inhibition of Stat3 signal transduction by PIAS3. Science.

[26] Liu B (1998). Inhibition of Stat1-mediated gene activation by PIAS1. Proc. Natl Acad. Sci. USA.

[27] Calvisi DF (2007). Mechanistic and prognostic significance of aberrant methylation in the molecular pathogenesis of human hepatocellular carcinoma. J. Clin. Invest..

[28] Puhr M (2010). SOCS-3 antagonises the proliferative and migratory effects of fibroblast growth factor-2 in prostate cancer by inhibition of p44/p42 MAPK signalling. Endocr. Relat. Cancer.

[29] Tang Q, Jiang J, Liu J (2015). CCR5 blockade suppresses melanoma development through inhibition of IL-6-Stat3 pathway via upregulation of SOCS3. Inflammation.

[30] Yeh JE, Frank DA (2016). STAT3-interacting proteins as modulators of transcription factor function: implications to targeted cancer therapy. ChemMedChem.

[31] Wang T (2015). Anxa2 binds to STAT3 and promotes epithelial to mesenchymal transition in breast cancer cells. Oncotarget.

[32] Wang Y (2014). GdX/UBL4A specifically stabilizes the TC45/STAT3 association and promotes dephosphorylation of STAT3 to repress tumorigenesis. Mol. Cell.

[33] Im JY (2016). DNA damage-induced apoptosis suppressor (DDIAS), a novel target of NFATc1, is associated with cisplatin resistance in lung cancer. Biochim. Biophys. Acta.

[34] Im JY (2016). DNA damage induced apoptosis suppressor (DDIAS) is upregulated via ERK5/MEF2B signaling and promotes beta-catenin-mediated invasion. Biochim. Biophys. Acta.

[35] Won KJ (2017). Stability of the cancer target DDIAS is regulated by the CHIP/HSP70 pathway in lung cancer cells. Cell Death Dis..

[36] Im JY (2018). DDIAS suppresses TRAIL-mediated apoptosis by inhibiting DISC formation and destabilizing caspase-8 in cancer cells. Oncogene.

[37] Zhang ZZ, Huang J, Wang YP, Cai B, Han ZG (2015). NOXIN as a cofactor of DNA polymerase-primase complex could promote hepatocellular carcinoma. Int. J. Cancer.

[38] Won KJ (2014). Human Noxin is an anti-apoptotic protein in response to DNA damage of A549 non-small cell lung carcinoma. Int. J. Cancer.

[39] Yuan ZL, Guan YJ, Chatterjee D, Chin YE (2005). Stat3 dimerization regulated by reversible acetylation of a single lysine residue. Science.

[40] Sobotta MC (2015). Peroxiredoxin-2 and STAT3 form a redox relay for H2O2 signaling. Nat. Chem. Biol..

[41] Wei J (2012). The ubiquitin ligase TRAF6 negatively regulates the JAK-STAT signaling pathway by binding to STAT3 and mediating its ubiquitination. PLoS ONE.

[42] Zhong Z, Wen Z, Darnell JE (1994). Stat3: a STAT family member activated by tyrosine phosphorylation in response to epidermal growth factor and interleukin-6. Science.

[43] Garcia R (2001). Constitutive activation of Stat3 by the Src and JAK tyrosine kinases participates in growth regulation of human breast carcinoma cells. Oncogene.

[44] Wang Z (2004). Mutational analysis of the tyrosine phosphatome in colorectal cancers. Science.

[45] LaForgia S (1991). Receptor protein-tyrosine phosphatase gamma is a candidate tumor suppressor gene at human chromosome region 3p21. Proc. Natl Acad. Sci. USA.

[46] Yu, C. et al. Circular RNA cMras inhibits lung adenocarcinoma progression via modulating miR-567/PTPRG regulatory pathway. *Cell Prolif.*10.1111/cpr.12610.e12610 (2019). 10.1111/cpr.12610 PMC6536402 31012177

[47] Gebbink MF (1995). Cell surface expression of receptor protein tyrosine phosphatase RPTP mu is regulated by cell-cell contact. J. Cell Biol..

[48] Sun PH, Ye L, Mason MD, Jiang WG (2012). Protein tyrosine phosphatase micro (PTP micro or PTPRM), a negative regulator of proliferation and invasion of breast cancer cells, is associated with disease prognosis. PLoS ONE.

[49] Sudhir PR (2015). Loss of PTPRM associates with the pathogenic development of colorectal adenoma-carcinoma sequence. Sci. Rep..

[50] Burgoyne AM (2009). Proteolytic cleavage of protein tyrosine phosphatase mu regulates glioblastoma cell migration. Cancer Res..

[51] Stevenson WS (2014). DNA methylation of membrane-bound tyrosine phosphatase genes in acute lymphoblastic leukaemia. Leukemia.

[52] Quintavalle C (2012). miR-221/222 overexpession in human glioblastoma increases invasiveness by targeting the protein phosphate PTPmu. Oncogene.

[53] Neel BG, Tonks NK (1997). Protein tyrosine phosphatases in signal transduction. Curr. Opin. Cell Biol..

[54] Zondag GC, Reynolds AB, Moolenaar WH (2000). Receptor protein-tyrosine phosphatase RPTPmu binds to and dephosphorylates the catenin p120(ctn). J. Biol. Chem..

[55] Mariner DJ, Davis MA, Reynolds AB (2004). EGFR signaling to p120-catenin through phosphorylation at Y228. J. Cell Sci..

[56] Alema S, Salvatore AM (2007). p120 catenin and phosphorylation: mechanisms and traits of an unresolved issue. Biochim. Biophys. Acta.

[57] Vichai V, Kirtikara K (2006). Sulforhodamine B colorimetric assay for cytotoxicity screening. Nat. Protoc..

