# Supplementary figures and images for: DDIAS promotes STAT3 activation by preventing STAT3 recruitment to PTPRM in lung cancer cells
Source: Oncogenesis. 2020 Jan 2;9(1):1. doi: 10.1038/s41389-019-0187-2 (PMC6949220; doi:10.1038/s41389-019-0187-2)

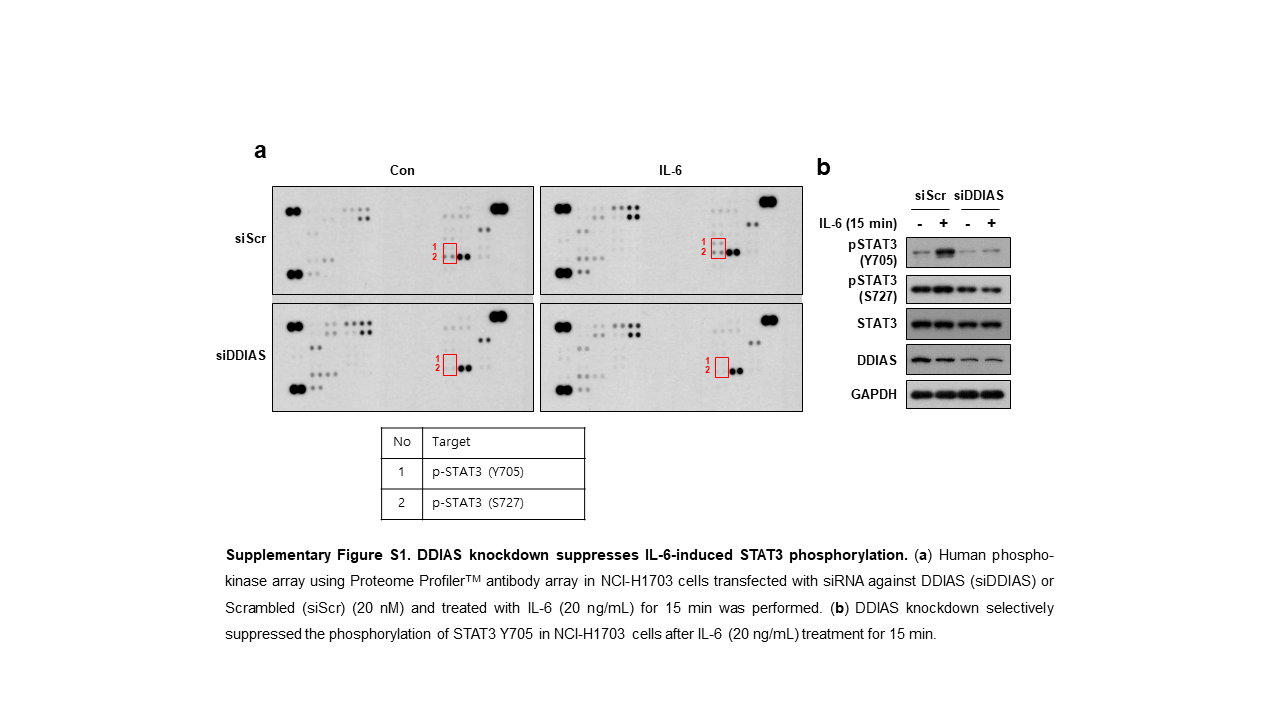

Supplement: Supplementary file 1 — Supplementary Figure S1. DDIAS knockdown suppresses IL-6-induced STAT3 phosphorylation [file 41389_2019_187_MOESM1_ESM.tif]

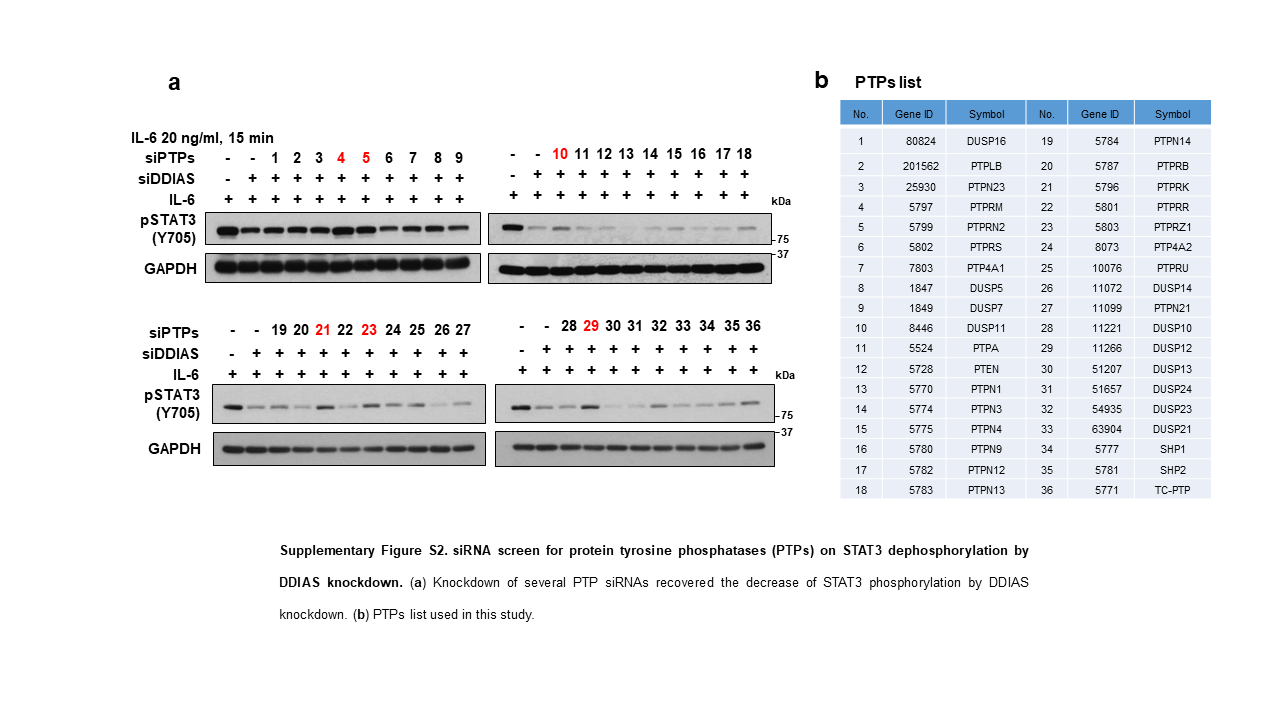

Supplement: Supplementary file 2 — Supplementary Figure S2. siRNA screen for protein tyrosine phosphatases (PTPs) on STAT3 dephosphorylation by DDIAS knockdown [file 41389_2019_187_MOESM2_ESM.tif]

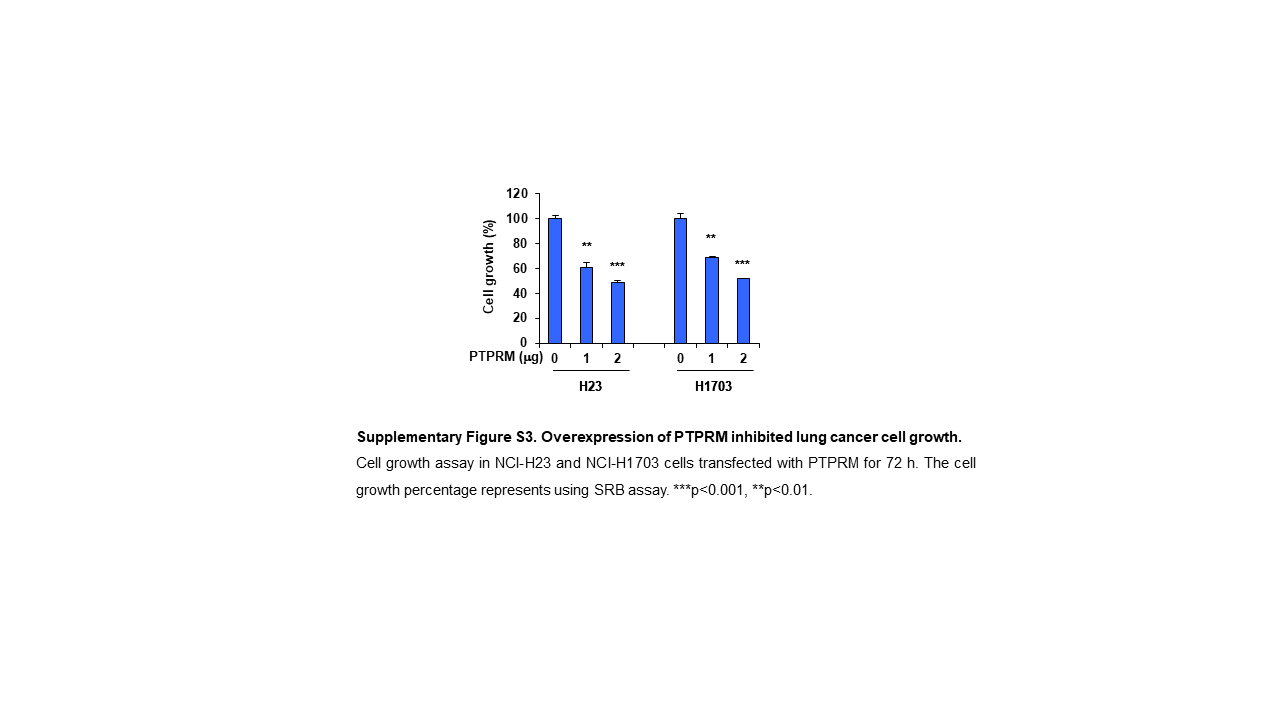

Supplement: Supplementary file 3 — Supplementary Figure S3. Overexpression of PTPRM inhibited lung cancer cell growth [file 41389_2019_187_MOESM3_ESM.tif]

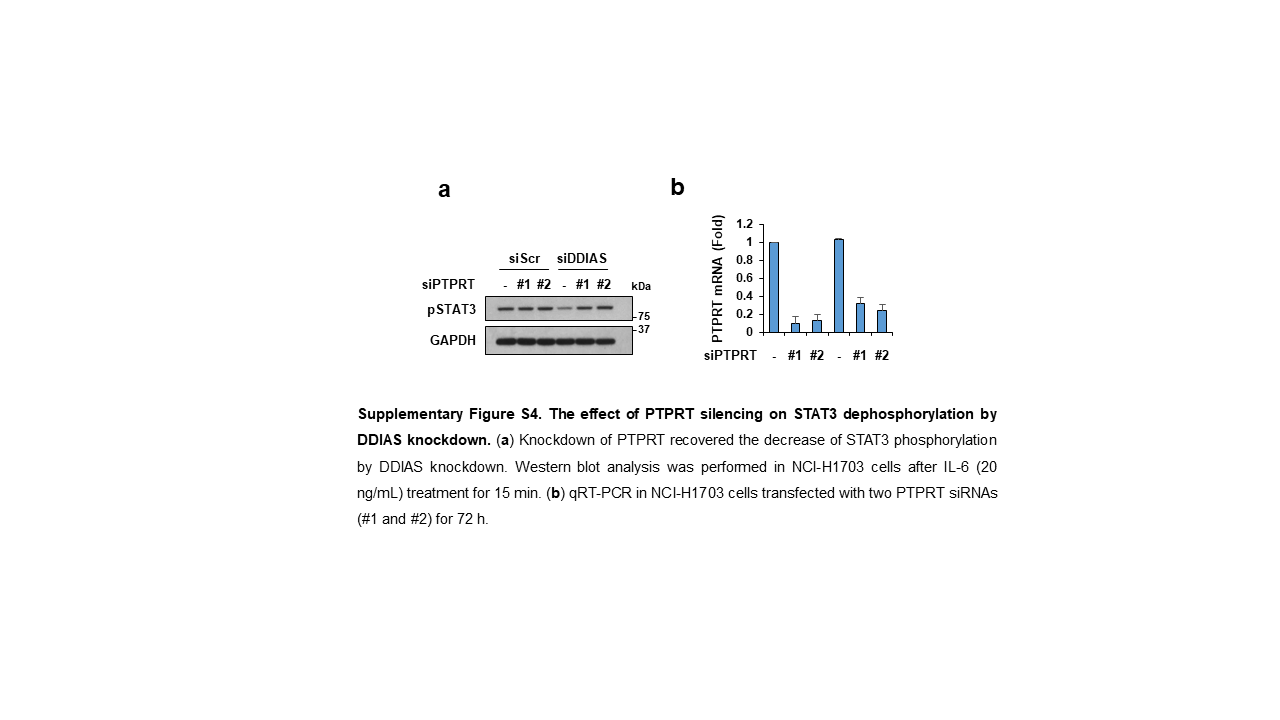

Supplement: Supplementary file 4 — Supplementary Figure S4. The effect of PTPRT silencing on STAT3 dephosphorylation by DDIAS knockdown [file 41389_2019_187_MOESM4_ESM.tif]

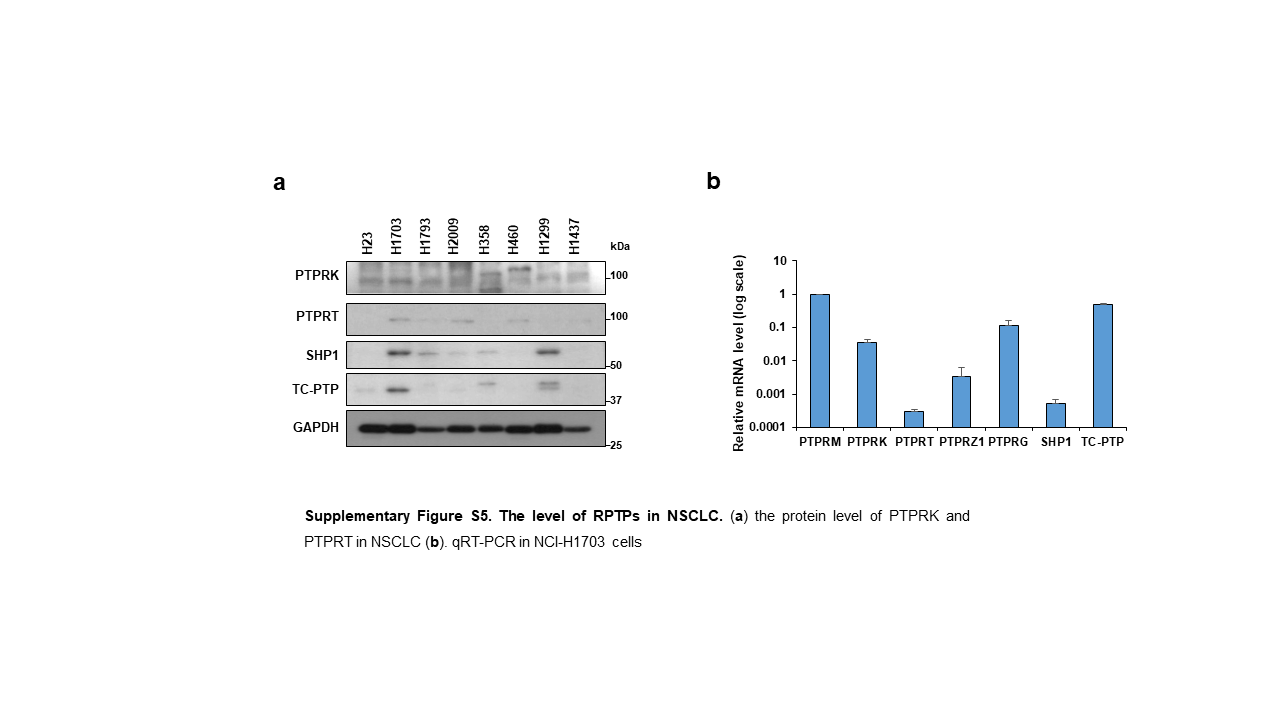

Supplement: Supplementary file 5 — Supplementary Figure S5. The level of RPTPs in NSCLC [file 41389_2019_187_MOESM5_ESM.tif]

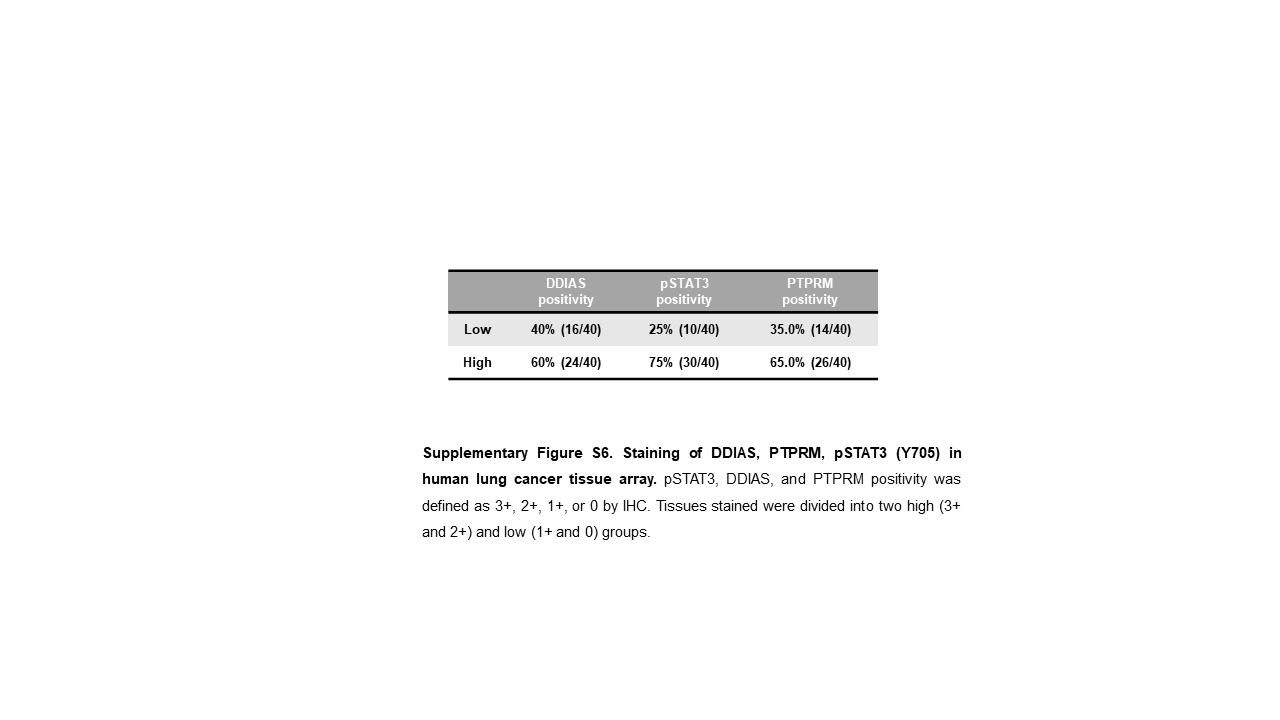

Supplement: Supplementary file 6 — Supplementary Figure S6. Staining of DDIAS, PTPRM, pSTAT3 (Y705) in human lung cancer tissue array [file 41389_2019_187_MOESM6_ESM.tif]
